# Mapping the Multidimensional Stress Burden in Nursing Students: A Systematic Review and Instrument‐Specific Meta‐Analysis

**DOI:** 10.1155/nrp/5514459

**Published:** 2026-07-12

**Authors:** Sibgha Fatima, Dragan Ilic, Michelle Lazarus, Zahra Aziz, Carol Tra Vu, Md Nazmul Karim

**Affiliations:** ^1^ School of Public Health and Preventive Medicine, Monash University, Melbourne, Australia, monash.ac.za; ^2^ Human Anatomy and Developmental Biology, Monash University, Melbourne, Australia, monash.ac.za

**Keywords:** DASS, instrument-specific meta-analysis, mental health, nursing students, perceived stress, prevalence, PSS, stress

## Abstract

Undergraduate nursing students experience stress as a multidimensional construct shaped by academic, clinical, psychosocial and contextual demands throughout training, placing them at increased risk of adverse mental health outcomes. Contemporary literature suggests that stress measures vary by instrument, reflecting distinct constructs (perceived vs. symptom‐based). This systematic review and meta‐analysis synthesised instrument‐specific pooled estimates of moderate‐to‐high stress among undergraduate nursing students and examined variation by training stage, country income level, geographical region, pandemic period and stress measurement approach. Electronic databases were searched for quantitative studies published between January 2010 and December 2025. Random‐effects meta‐analyses were conducted separately for studies using the Perceived Stress Scale (PSS), reflecting perceived stress, and the Depression Anxiety Stress Scale (DASS) stress subscale, reflecting affective stress symptoms. Subgroup, sensitivity and meta‐regression analyses were undertaken to explore heterogeneity. Forty‐two studies involving 19,655 students from 21 countries met the inclusion criteria, with 27 studies contributing to prevalence synthesis and 18 to pooled mean scores. Given the very high heterogeneity and the non‐equivalence of instruments, an overall pooled prevalence of 49.98% was calculated only as a descriptive summary. Instrument‐specific analyses indicated a substantial prevalence of perceived stress in PSS studies (74.37%) and symptom‐based stress in DASS studies (30.46%), while mean scores across both scales suggested moderate stress levels. However, substantial unexplained heterogeneity persisted, indicating the influence of unmeasured contextual, psychosocial and methodological factors across studies. These findings emphasise the wide range and variability of reported stress prevalence, suggesting that a pooled estimate may not adequately capture the underlying complexity. Overall, estimates are presented according to instrument‐defined constructs, and cross‐instrument comparisons should be interpreted with caution, highlighting the need for measurement‐aware, context‐sensitive research and interventions.

## 1. Background

Stress among undergraduate nursing students has emerged as a major global concern with significant implications for student well‐being, educational outcomes and the sustainability of the nursing workforce [1, 2]. In the context of increasing global demands on healthcare systems, excessive psychological distress during health professions education and training is particularly concerning [3, 4]. The consequences of elevated stress among nursing students extend beyond transient discomfort. Literature documents associations between student stress and adverse outcomes, including poorer mental health, risk of burnout, impaired concentration and learning, higher intentions to leave the profession, and suicidal ideation—all of which have implications for individual well‐being and future patient safety [5–9].

Nursing education is inherently demanding, combining intensive academic curricula with emotionally and physically challenging clinical training [10, 11]. Students are required to acquire complex theoretical knowledge, demonstrate competence in high‐stakes clinical environments, and adapt to professional socialisation, including learning professional norms, communication skills, teamwork behaviours, and the standards of accountability expected in healthcare practice. This dual academic–clinical workload places prelicensure nursing students at elevated risk of perceived psychological strain, with multiple syntheses of the literature characterising stress in this population as moderate‐to‐high and widely prevalent across countries and curricula [12]. The distress is particularly pronounced during disruptive periods such as the COVID‐19 era, when academic and clinical demands intensified [13].

**FIGURE 1:**
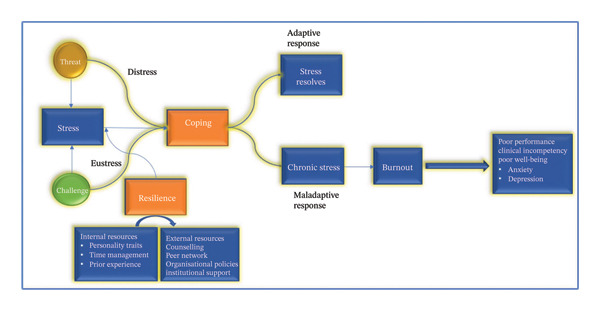
A conceptual model of stress trajectory.

Stress is described as a psychological and physiological response that arises when individuals perceive environmental demands exceed their adaptive capacity [14, 15]. Stress is triggered by stimuli called stressors. According to the transactional model of stress and coping, a stressor can be appraised either as a challenge or a potential for motivation that leads to eustress (positive stress). However, a stressor perceived as a threat is a potential for harm or loss and results in distress (negative stress) and adverse outcomes [15, 16]. Students’ stress perception varies depending upon the internal and external resources available to them, including prior experience, resilience, coping strategies, personality traits, time management skills, and institutional support programmes. According to Cooper [17], when these resources are perceived as inadequate, students may become trapped in a cause‐re‐cause cycle in which stressors trigger stress, and resulting stress further amplifies the perceived intensity of the stressors, leading to an ongoing stress experience [18]. Prolonged exposure to such chronic stress has been associated with burnout, depression and anxiety [19]; reduced academic performance [5]; and increased risk of substance use [20], attrition [21] and suicidal ideation [22], ultimately compromising student well‐being [23]. This can lead to significant deterioration of mental health [24] as described in the conceptual model of stress trajectory (Figure 1).

**FIGURE 2:**
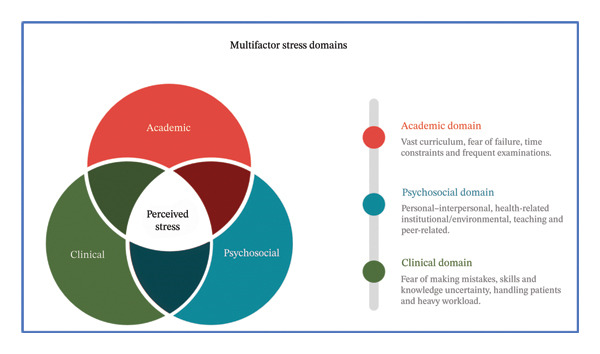
Conceptual model of nursing students’ stress domains.

Stress during nurse training is multidetermined and commonly situated within three overlapping domains: academic, clinical and psychosocial domains [25–29]. A conceptual model illustrating these three overlapping domains is presented in Figure 2. These domains represent potential contributors that shape stress appraisals during nursing training and are consistently reported in literature [9, 30]. Together they help contextualise why many nursing students report sustained levels of perceived stress and psychological distress during their educational tenure [28, 31]. High and sustained stress during nursing education can negatively influence students’ well‐being and readiness for professional practice [32].

Contemporary literature conceptualises stress as a multidimensional construct, reflecting different theoretical and measurement perspectives [15, 33]. From a cognitive‐appraisal perspective [15], a framework commonly operationalised by instruments such as the Perceived Stress Scale (PSS) [34]. PSS is a self‐report measure designed to assess the degree to which life situations are appraised as stressful and characterised by unpredictability, uncontrollability and overload [34, 35]. In contrast, symptom‐based models conceptualise stress through observable emotional and somatic manifestations such as tension, irritability and physiological arousal, captured by tools like the Depression Anxiety Stress Scale (DASS) [36]. DASS includes a distinct stress subscale alongside depression and anxiety components [37, 38]. Additionally, stress may be examined as a domain‐specific construct, referring to stress responses arising within particular contexts, that is, academic, occupational, or clinical environments, recognising that stressors, appraisals and outcomes vary across settings [18, 39]. Together, these perspectives underscore that stress is not a unitary phenomenon but a complex construct shaped by cognitive appraisal, symptomatic expression and contextual domain. Accordingly, stress is treated as a dynamic, instrument‐defined psychological burden rather than a diagnostic entity, capturing perceived demands and stress responses that are conceptually distinct from anxiety‐related disorders [40, 41]. Importantly, none of the studies have comprehensively synthesised global stress burden by exploring variability across study contexts and measurement approach, while explicitly accounting for the multidimensional nature of stress.

Over the past decade, a growing body of international literature has examined stress among undergraduate nursing students across diverse geographical and socio‐economic contexts [12, 42, 43]. Recent studies from Asia, Europe, the Middle East, Africa, and the Americas consistently report high levels of stress among undergraduate nursing students, although prevalence estimates vary substantially across settings [44–46]. Despite the growing number of systematic reviews reporting pooled stress prevalence among nursing students, existing syntheses have typically combined results across heterogeneous stress‐measuring instruments, treating stress as a unitary outcome, and thereby obscuring both the multidimensional nature of stress and the distinct constructs captured by each tool [30, 46–49]. To align the evidence synthesis with contemporary stress theory and to avoid assuming cross‐instrument equivalence, the present review derives pooled estimates separately for each measurement instrument, that is, PSS and DASS Stress subscale, ensuring that the resulting estimates remain conceptually coherent and interpretable. A systematic review that is explicitly grounded in these theoretical and methodological considerations is therefore warranted to provide a more robust and meaningful evidence base.

The primary aim of this systematic review is to synthesise instrument‐specific estimates of moderate‐to‐high stress across PSS and DASS Stress subscales among undergraduate nursing students and examine how reported prevalence varies across study contexts and measurement approach. By treating these instruments as non‐interchangeable operationalisations of stress, the review explicitly acknowledges the multidimensional nature of the construct and avoids conflating conceptually distinct measurement approaches. The secondary aim is to pool mean stress scores across studies using the PSS and DASS, thereby complementing prevalence estimates with continuous outcome synthesis and enabling a more nuanced appraisal of stress burden within each instrument. Importantly, the review is grounded in contemporary stress theory, positioning it as methodologically additive rather than merely an update of prior syntheses.

## 2. Methods

### 2.1. Protocol and Reporting

This systematic review and meta‐analysis were conducted and reported in accordance with the Preferred Reporting Items for Systematic Reviews and Meta‐Analyses (PRISMA 2020) guidelines. The review protocol was prospectively registered on PROSPERO 2026 CRD420251266378.

### 2.2. Research Question

We defined our research question based on PEO—Population: undergraduate nursing students, Exposure: stressors/sources of stress. Outcome: stress measured through validated stress instruments. We defined our research question as What is the distribution of stress among undergraduate nursing students, as quantified by validated instruments such as the PSS and the DASS‐Stress subscale, which capture different dimensions of the multidimensional stress construct?

### 2.3. Search Strategy

We performed a comprehensive electronic search of Ovid MEDLINE, Embase Classic, Emcare and PsycINFO from January 2010 to December 2025. The search combined subject headings (MeSH/Emtree where applicable) and free‐text terms mapped to three core concepts: (A) stress, (B) prevalence/epidemiology and (C) nursing students. Controlled vocabulary and text words used included, for example: Concept A (stress): ‘“Stress”[MeSH]’ OR ‘psychological’ OR ‘emotional exhaustion’ OR ‘financial stress’ OR ‘subjective stress’ OR ‘time pressure’ OR ‘perceived stress∗’ OR ‘student stress∗’ OR ‘academic stress∗’ OR ‘psychologic∗ stress∗’, Concept B (prevalence): ‘incidence [MeSH]’ OR ‘prevalence [MeSH]’ OR ‘incidence’ OR ‘prevalence’ OR ‘epidemiology’ OR ‘distribution’ OR ‘proportion’ OR ‘magnitude’, Concept C (students): ‘“Students, Nursing”[MeSH]’ OR ‘nursing student∗’ OR ‘undergraduate nursing’ OR ‘baccalaureate nursing’ (we also included medical student terms to capture comparative/mixed studies and then extracted nursing‐specific data where available). These three concept blocks were combined with Boolean operator ‘AND’. Searches were limited to the English language and the specified date range. The full search strategy for Ovid Medline (and adapted strings for all other databases) is provided in Supporting Appendix A Table S1. Reference lists of included studies and relevant reviews were hand‐searched to identify additional records.

### 2.4. Study Selection

Studies that met the inclusion criteria were included in the review. Eligible studies reported either prevalence (proportion) or mean scores of perceived, academic, psychological, or student stress measured using validated instruments such as the PSS, DASS‐Stress subscale, or Student Stress Inventory in undergraduate nursing students only. Studies that included mixed cohorts of health‐professional students were eligible only when nursing‐specific data were reported separately. Observational designs (cross‐sectional or cohort), published between January 2010 and December 2025, were included to capture stress estimates within contemporary nursing education contexts characterised by evolving curricula, increasing clinical demands and widespread use of standardised stress tools across educational environments. Restriction to English‐only studies ensures accurate interpretation and maintains feasibility.

We excluded studies that (1) did not report extractable prevalence or mean data and studies where nursing student results could not be disaggregated from the overall sample of mixed population; (2) used nonvalidated stress measurement tools; (3) were reviews, commentaries, editorials, case reports, or conference abstracts without full data; or (4) were duplicate reports of the same dataset (the most complete report was retained).

**FIGURE 3:**
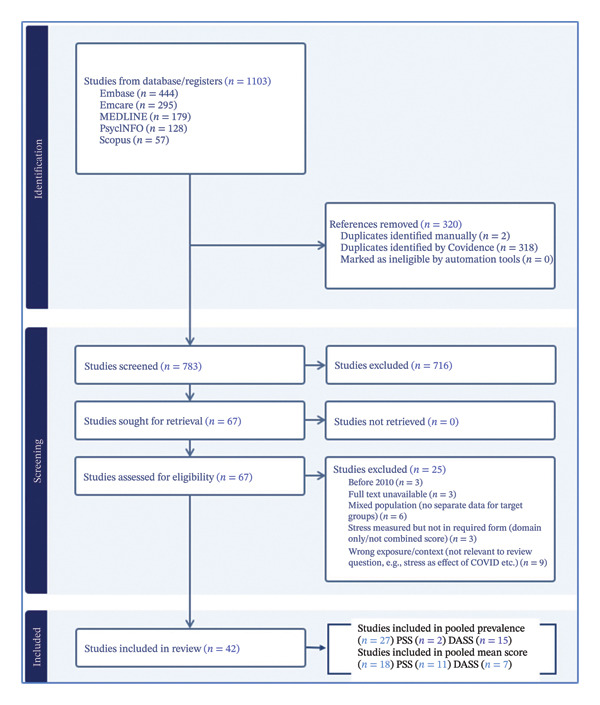
Identification of studies via the databases. PRISMA (Preferred Reporting Items for Systematic Reviews and Meta‐Analysis) 2020 flowchart of the peer‐reviewed study selection process.

All records identified through the search were imported into Covidence (Covidence Ltd) for management. Following automated and manual deduplication, two reviewers (S.F. and C.T.V.) independently screened titles and abstracts against the predefined eligibility criteria. Full texts of potentially relevant studies were then retrieved and assessed independently by the same reviewers. Any discrepancies at either stage were resolved through discussion, with unresolved disagreements adjudicated by a third reviewer (M.N.K.). The study selection process is presented in the PRISMA 2020 flow diagram (Figure 3).

### 2.5. Data Extraction

The first reviewer (S.F.) conducted the initial data extraction for all included studies. The second reviewer (C.T.V.) verified each extraction against the original studies. A piloted data extraction form was used. The form was tested on a subset of studies to refine variable definitions, ensure clarity, and standardise reviewer interpretation before full extraction. Extracted items included the author, year, country, study design, setting, sample size, participant characteristics (mean age and sex distribution), instrument used to measure stress, instrument cut‐offs and scoring (including the threshold used to define ‘stress levels as mild, moderate, high or extremely high’ where applicable), prevalence estimates (proportions and reported 95% confidence intervals [CIs]), mean scores and standard deviations (SDs) (when reported), and additional contextual variables included training stage, year of study and COVID‐19 period, mean age and gender distribution. Different PSS versions were handled separately because their score ranges and cut‐offs are not interchangeable. Studies using instruments other than the DASS or PSS, that is, SINS‐CH and Academic Stress Scales, were synthesised narratively when fewer than three studies were available per tool and were excluded from the meta‐analyses.

### 2.6. Outcome Definition and Harmonisation of Stress Measures

Given the conceptual and psychometric differences between stress instruments, outcomes were defined and synthesised according to the specific tool used, without assuming cross‐instrument equivalence. Studies were grouped by instrument, with separate meta‐analyses conducted for the PSS and the DASS subscale. As established in the psychometric literature, the PSS and DASS‐Stress subscale measure distinct facets of stress, that is, cognitive appraisal versus physiological/affective symptoms, ensuring their treatment as separate constructs in this review [34, 36, 50]. For each tool, the primary outcome was the prevalence of instrument‐defined moderate‐to‐high stress, operationalised using severity thresholds recommended in the respective scale manuals and applied consistently across studies. Because severity thresholds are instrument‐specific and not psychometrically interchangeable, pooled estimates were interpreted as reflecting the instrument‐defined burden of moderate‐to‐high stress (stress perception vs. affective symptoms) rather than a universal prevalence of stress.

For PSS studies, prevalence was calculated by summing the proportions reporting moderate and high stress, reflecting stress perception. Prevalence estimates were pooled using study‐specific thresholds for moderate‐to‐high stress as defined by the respective PSS versions (PSS‐10 and PSS‐29). For DASS studies, estimates were derived by combining moderate, severe, and extremely severe stress categories, reflecting affective symptom burden. The moderate threshold was selected as the lowest level above normative stress to capture functionally meaningful stress across instruments; however, differences in scale construction limit direct equivalence. Accordingly, pooled estimates were interpreted as descriptive summaries of instrument‐defined stress burden, rather than as estimates of a single underlying construct.

For the secondary outcome, mean stress scores with corresponding SDs were extracted directly from each study as reported by the authors. Pooled mean stress scores were calculated separately for each stress assessment tool to preserve scale interpretability and avoid distortion arising from differences in scoring ranges and severity thresholds across tools. Studies reporting continuous stress outcomes using the PSS or the DASS Stress subscale were considered eligible for quantitative synthesis of mean stress scores. Studies employing other stress measurement instruments, that is, SINs‐CN, Academic Stress Scale or alternative versions with insufficient representation, were included in the review but were not pooled quantitatively due to an inadequate number of studies (< 3). These studies were synthesised narratively.

### 2.7. Risk of Bias Assessment

Study quality was assessed independently by two reviewers (S.F. and C.T.V.) using the modified Newcastle–Ottawa Scale (NOS) for cross‐sectional studies following the adaptation by Herzog et al. [51]. The scale includes a 10‐item adaptation appropriate for observational survey designs. The adapted NOS evaluates selection (representativeness of the sample and sample size justification), comparability (adjustment for key confounders where applicable) and outcome (validity/reliability of outcome measurement and statistical reporting). Scores were categorised as low risk (7–10), moderate risk (5–6) and high risk (0–4). Discrepancies were resolved by discussion and consensus.

### 2.8. Data Synthesis and Statistical Analysis

All statistical analyses were performed using Stata (Version 19; StataCorp, College Station, TX, USA). Pooled prevalence estimates with 95% CIs were calculated for moderate‐to‐high stress, as defined a priori based on instrument‐specific severity classifications, while continuous stress outcomes were extracted as reported mean stress scores with corresponding SDs. Out of 42 included studies, where studies reported both prevalence and mean stress scores, each outcome was analysed independently in its respective meta‐analysis. A total of 27 studies contributed to the pooled prevalence analyses. Of these, 15 studies were synthesised in the DASS‐based prevalence meta‐analysis, and 12 studies were included in the PSS‐based prevalence meta‐analysis. For the secondary outcome, 18 studies contributed to the pooled mean stress analyses, comprising 11 PSS‐based studies and 7 DASS‐based studies. Stress outcomes measured using other instruments were summarised using narrative synthesis to preserve interpretability and avoid inappropriate pooling.

Between‐study heterogeneity was assessed using Cochran’s Q test and quantified using the *I* ^2^ statistic. Given the observational nature of the included prevalence studies and the variability in measurement instruments and study contexts, high *I* ^2^ values were anticipated.

Prespecified subgroup analyses were conducted to explore potential sources of heterogeneity based on stress assessment tool, country income classification, geographical regions, COVID period, training stage and study quality. Sensitivity analyses were performed by excluding studies at high risk of bias and by sequentially omitting individual studies to assess the robustness of pooled prevalence estimates.

Potential publication bias was evaluated through visual inspection of funnel plots and formally assessed using Begg’s rank correlation test, with *p* < 0.05 considered indicative of small‐study effects.

Meta‐regression analysis was conducted to explore potential sources of between‐study heterogeneity in pooled stress prevalence estimates. A random‐effects model was applied using the REML estimator, with study‐level covariates selected a priori, including sample size, year of publication, stress assessment tool, geographical region, country income classification, training status, COVID period and study quality score. Regression coefficients (β) with corresponding standard errors and *p*‐values were reported to assess associations between covariates and prevalence estimates. The proportion of between‐study variance explained by included covariates was evaluated using the adjusted *R* ^2^ statistic. Findings from meta‐regression were interpreted cautiously due to the ecological nature of the analysis.

## 3. Results

A total of 1103 records were identified through database searching. After removing duplicates (*n* = 320), 783 records were screened by title and abstract. Full texts of 67 articles were assessed for eligibility, of which 42 studies met the inclusion criteria and were included in the meta‐analyses.

### 3.1. Study and Population Characteristics

A total of 42 studies published between 2010 and 2025 were included, encompassing 19,655 undergraduate nursing students from 21 countries across low‐income (Ethiopia and Nepal), middle‐income (India, China, Indonesia, Turkey, Vietnam, Morocco, Nigeria, Egypt, the Philippines, Pakistan, Iran and Peru) and high‐income settings (Australia, the United States, Saudi Arabia, Korea, the United Kingdom, Oman and the UAE). All the included studies were cross‐sectional in design, with sample sizes ranging from 74 to 1572 participants, and reported response rates varying between 19% and 100%. Most participants were in years 1–4 of training, spanning both preclinical and clinical stages. Data were collected across pre‐COVID (*n* = 6), during COVID (*n* = 12), and post‐COVID periods (*n* = 24). Stress was measured predominantly using the PSS (10‐, 14‐, and 29‐item versions; *n* = 19) and the DASS (21‐ and 42‐item versions; *n* = 13), with a few studies using other validated scales like SINS‐CN and the Academic Stress Scale. The studies employed convenience sampling (*n* = 27), stratified or proportionate stratified random sampling (*n* = 10), simple random sampling (*n* = 3), and total/multistage sampling (*n* = 2) (Table 1). Overall, the included studies represent a geographically and methodologically diverse population, providing a robust basis for pooled prevalence and mean stress score meta‐analysis. Of the 42 included studies, 27 contributed to the pooled prevalence meta‐analysis, while 18 were included in the pooled mean stress score meta‐analysis, with some studies contributing to both analyses depending on the reported outcome.

**TABLE 1:** Study and population characteristics.

Study	Country	Country income status	Setting	Study year	Study design	Sampling method	Sample	Response rate	Academic level	Stage of training	COVID‐19 period	Tool used	NOS score
Ezo et al. [46]	Ethiopia	Low income	University	2024	Cross‐sectional	Proportionate stratified random sampling	422	100	Years 2–4	Clinical	Post‐COVID	PSS‐29	Low
Asif et al. [52]	Pakistan	Middle income	Nursing schools	2019	Cross‐sectional	Simple random sampling	220	90	Years 1–4	Mixed	Pre‐COVID	PSS‐10	Moderate
Farber and Amorim [53]	United States	High income	University	2023	Cross‐sectional	Convenience sampling	111	94	Years 2–4	Clinical	Post‐COVID	PSS‐10	Low
Pawar et al. [54]	India	Middle income	Institution	2021	Cross‐sectional	Convenience sampling	300	100	Years 1–4	Mixed	In‐COVID	PSS‐10	Moderate
Andargeery et al. [55]	Saudi Arabia	High income	College	2023	Cross‐sectional	Convenience sampling	237	96.3	Years 1–4	Mixed	Post‐COVID	DASS‐21	Low
Karithiga et al. [56]	India	Middle income	College	2023	Cross‐sectional	Simple random sampling	291	100	Years 1–4	Mixed	Post‐COVID	PSS‐10	Low
Rammouz et al. [57]	Morocco	Middle income	University	2021	Cross‐sectional	Convenience sampling	413	91.3	Years 1–3	Mixed	In‐COVID	PSS‐10	Low
Smith et al. [58]	United States	High income	College	2020	Cross‐sectional	Convenience sampling	490	19	Years 1–4	Mixed	In‐COVID	PSS‐10	Low
Baruah et al. [59]	India	Middle income	College	2021	Cross‐sectional	Convenience sampling	214	NR	Years 1–4	Mixed	In‐COVID	DASS‐21	High
Albikawi [60]	Saudi Arabia	High income	University	2022	Cross‐sectional	Convenience sampling	115	NR	Years 1–4	Mixed	In‐COVID	DASS‐21	Moderate
Ngoc and Tuan [61]	Vietnam	Middle income	College	2020	Cross‐sectional	Stratified random sampling method	300	100	Years 1–3	Mixed	In‐COVID	DASS‐21	High
Resano et al. [62]	Philippines	Middle income	College	2020	Cross‐sectional	Proportionate stratified random sampling	249	37.4	Years 1–4	Mixed	In‐COVID	PSS‐10	Low
Sarkar et al. [63]	India	Middle income	Tertiary care centre	2022	Cross‐sectional	Proportionate stratified random sampling	201	98.5	Years 1, 2 and 4	Nursing students	In‐COVID	PSS‐10	Low
Maqbali et al. [64]	Oman	High income	College	2021	Cross‐sectional	Convenience sampling	548	NR	Years 1–4	Mixed	In‐COVID	PSS‐10	Moderate
Helenpuii 2024 [65]	India	Middle income	College	2024	Cross‐sectional	Convenience sampling	201	NR	Years 1–4	Mixed	Post‐COVID	DASS‐21	Moderate
Dogham et al. [66]	Egypt	Middle Income	College	2024	Cross‐sectional	Convenience sampling	1010	NR	Years 1–4	Mixed	Post‐COVID	PSS‐29	Low
Mohamed et al. [43]	Saudi Arabia	High income	University	2023	Cross‐sectional	Convenience sampling	113	100	Years 1–4	Mixed	Post‐COVID	PSS‐10	Low
El‐Ashry et al. [67]	Egypt	Middle income	College	2023	Cross‐sectional	Proportionate stratified random sampling	1572	95.97	Years 1–4	Mixed	Post‐COVID	DASS‐21	Low
Cao et al. [68]	China	Middle income	College	2023	Cross‐sectional	Proportionate stratified random sampling	1308	NR	Years 1–2	Preclinical	Post‐COVID	PSS‐14	Moderate
Komariah et al. [69]	Indonesia	Middle income	University	2023	Cross‐sectional	Total sampling	260	86.67	Years 1	Preclinical	Post‐COVID	PSS‐10	Moderate
Sarfika et al. [70]	Indonesia	Middle income	University	2024	Cross‐sectional	Proportionate stratified random sampling	250	100	Years 1–4	Mixed	Post‐COVID	DASS‐21	Low
Abaribe et al. [71]	Nigeria	Middle income	University	2025	Cross‐sectional	Multistage sampling	204	100	Level 3, 4 and 5	Clinical	Post‐COVID	PSS‐10	Moderate
Bernier Carney et al. [72]	United States	High income	University	2023	Cross‐sectional	Convenience sampling	295	NR	Years 1–4	Mixed	Post‐COVID	PSS‐10	High
Lee [73]	Korea	High income	College	2013	Cross‐sectional	Convenience sampling	518	100	Years 1–4	Mixed	Pre‐COVID	PSS‐10	Moderate
Diaz‐Godiño et al. [74]	PERU	Middle income	Nursing schools	2018	Cross‐sectional	Convenience sampling	1193	100	Years 1–4	Mixed	Pre‐COVID	DASS‐21	Moderate
Onieva‐Zafra et al. [75]	Spain	High income	University	NR	Cross‐sectional	Convenience sampling	192	85.3	Years 2–4	Mixed	NR	PSS‐14	Low
Kalkan Uğurlu et al. [76]	Turkey	Middle income	University	2021	Cross‐sectional	Convenience sampling	411	93.83	Years 1–4	Mixed	In‐COVID	DASS‐42	Moderate
Alsolais et al. [77]	Saudi Arabia	High income	University	2021	Cross‐sectional	Convenience sampling	492	46.50	Years 2–4	Clinical	In‐COVID	DASS‐21	Moderate
Stanton et al. [78]	Australia	High income	University	2017	Cross‐sectional	Convenience sampling	500	74.6	Years 1–4	Mixed	Pre‐COVID	DASS‐21	Low
Devi et al. [79]	Indonesia	Middle income	University	2016	Cross‐sectional	Convenience sampling	336	94.1	Years 1–4	Mixed	Pre‐COVID	DASS‐42	Moderate
Yesilot et al. [80]	Turkey	Middle income	University	2021	Cross‐sectional	Convenience sampling	366	38.6	Years 1–4	Mixed	In‐COVID	DASS‐21	Low
El Madani et al. [81]	Morocco	Middle income	Institution	2018	Cross‐sectional	Convenience sampling	405	74.1	Years 1–3	Mixed	Pre‐COVID	PSS‐10	Moderate
Al Maqbali et al. [82]	KSA, Oman, United Kingdom and UAE	High income	College and University	2021	Cross‐sectional	Convenience sampling	918	87.1	Years 1–4	Mixed	In‐COVID	PSS‐10	Moderate
Hemme and Renolita [83]	Indonesia	Middle income	University	NR	Cross‐sectional	Convenience sampling	157	60.3	Years 2–4	Clinical	In‐COVID	DASS‐42	Moderate
Stubin et al. [84]	United States	High income	University	2023	Cross‐sectional	Convenience sampling	989	20.1	Years 1–4	Mixed	Post‐COVID	DASS‐21	High
Yigit et al. [85]	Turkey	Middle income	University	2024	Cross‐sectional	Convenience sampling	544	97	Years 1–4	Mixed	Post‐COVID	DASS‐21	Moderate
Koirala et al. [45]	Nepal	Low income	University	NR	Cross‐sectional	Probability‐proportionate random stratified sampling	317	100	Years 2–4	Clinical	In‐COVID	PSS‐10	Low
Diaz et al. [86]	United States	High income	Institution	2021	Cross‐sectional	Convenience sampling	74	31.5	Years 1–4	Mixed	In‐COVID	PSS‐10	Low
Ayaz‐Alkaya et al. [87]	Turkey	Middle income	University	2023	Cross‐sectional	Convenience sampling	808	82	Years 1–4	Mixed	Post‐COVID	PSS‐29	Low
Amr et al. [88]	Egypt	Middle income	College	2009	Cross‐sectional	Stratified random sampling from each year	373	92.8	Years 1–4	Mixed	Pre‐COVID	PSS‐14	Low
Smith and Yang [89]	China	Middle income	Nursing schools	2013	Cross‐sectional	Convenience sampling	1538	97	Years 1–4	Mixed	Pre‐COVID	SINS‐CN‐43	Moderate
Nazari et al. [90]	Iran	Middle income	University	2024	Cross‐sectional	Convenience sampling	200	95.2	Years 1–4	Mixed	Post‐COVID	Academic Stress Scale‐27	Low

### 3.2. Study Quality

The quality of included studies was assessed using the NOS, scored out of 10, with studies scoring 7–10 classified as low risk of bias, 5–6 as moderate risk of bias and < 5 as high risk of bias. Based on the quality score of NOS, 15 studies were rated as low risk of bias, 17 studies as moderate risk of bias, and 5 studies as high risk of bias (Table 1 and Supporting Appendix A Table S2).

### 3.3. Results of Meta‐Analysis

A total of 27 studies comprising 11,424 participants from 13 countries were included in the overall prevalence meta‐analysis. Using a random‐effects model, the pooled prevalence of stress was 49.98% (95% CI: 39.05–60.91) (Supporting Appendix A Table S3). Notably, the overall pooled prevalence is presented strictly as a descriptive summary only, as combining psychometrically non‐equivalent instruments (PSS and DASS) limits interpretability. To reflect the non‐interchangeability of stress measurement instruments, pooled prevalence analyses were conducted separately for studies using the PSS and the DASS Stress subscale and should not be interpreted as directly comparable measures of prevalence.

### 3.4. Instrument‐Specific Pooled Prevalence With PSS and DASS Stress Subscale Studies

**FIGURE 4:**
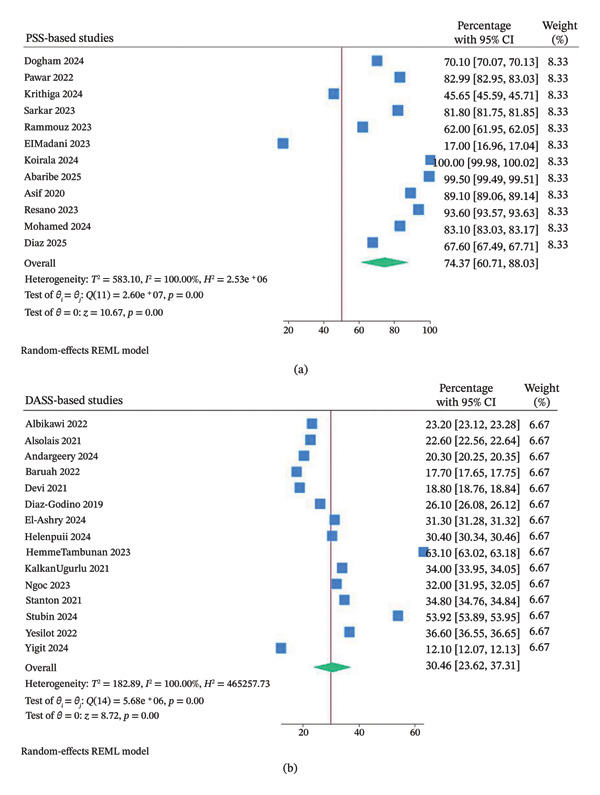
(a) Instrument‐specific pooled prevalence of moderate‐to‐high stress among nursing students within PSS studies. (b) Instrument‐specific pooled prevalence of moderate‐to‐high stress among nursing students within DASS Stress subscale studies.

Across 12 PSS‐based studies (**43, 46, 47, 51, 52, 54, 55, 60, 69, 74 and 75**), the pooled prevalence of moderate‐to‐high stress was 74.37% (95% CI: 61.29–87.45), with substantial heterogeneity (*I* ^2^ = 100%, *τ* ^2^ = 534.51, *p* < 0.001) (Figure 4a). The pooled prevalence from 15 DASS‐based studies (**45, 48, 49, 50, 53, 56, 63, 65, 66, 67, 44, 68, 71, 72 and 73**) was 30.46% (95% CI: 23.85–37.07), accompanied by high heterogeneity (*I* ^2^ = 100%, *τ* ^2^ = 170.70, *p* < 0.001) (Figure 4b). Analyses were conducted separately for each instrument, and no statistical comparison between PSS and DASS estimates was performed.

### 3.5. Instrument‐Specific Pooled Mean

**FIGURE 5:**
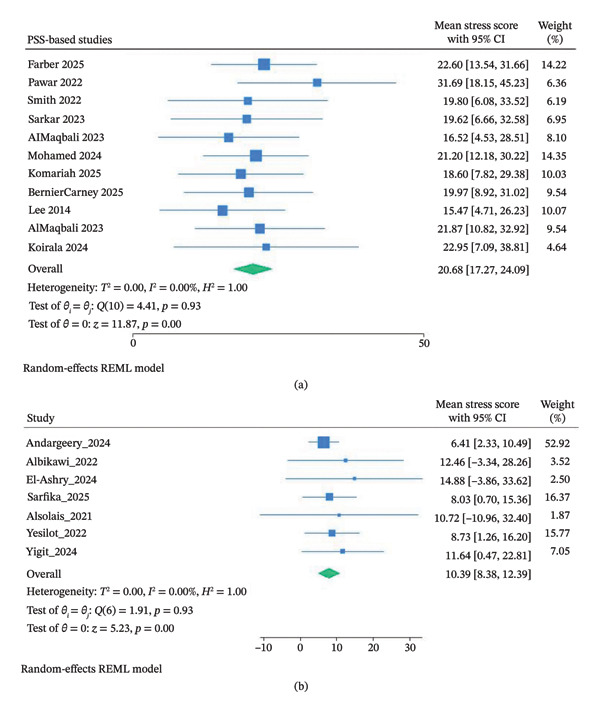
(a) Instrument‐specific pooled mean within studies using PSS. (b) Instrument‐specific pooled mean within studies using DASS.

Across 11 PSS‐based studies (**44, 12, 30, 52, 47, 55, 58, 61, 62, 70 and 74**) (score range: 0–40), the pooled mean stress score was 20.93 (95% CI: 18.52–23.33), with substantial heterogeneity (*I* ^2^ = 99.39%, *τ* ^2^ = 16.43, *p* < 0.001) (Figure 5a). For the 7 DASS‐based studies (**45, 49, 56, 59, 66, 68 and 73**) (score range: 0–42), the pooled mean was 10.39 (95% CI: 8.38–12.39), accompanied by high heterogeneity (*I* ^2^ = 99.20%, *τ* ^2^ = 7.16, *p* < 0.001) (Figure 5b). These results reflect instrument‐specific stress severity and are not directly comparable across scales.

### 3.6. Instrument‐Specific Subgroup Meta‐Analyses

**FIGURE 6:**
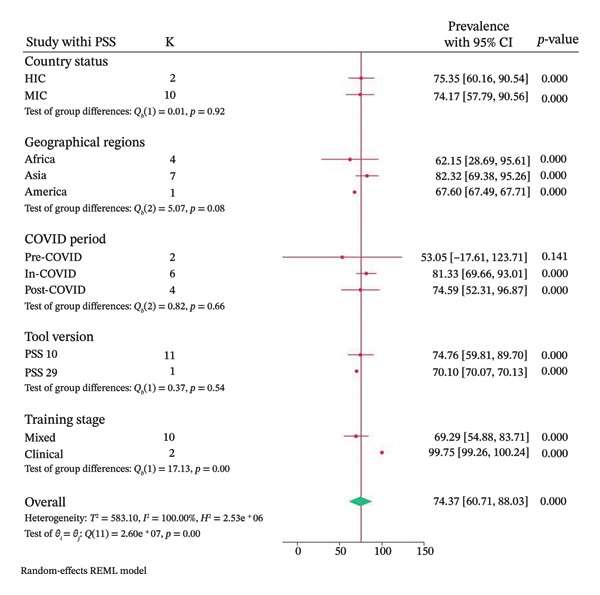
Instrument‐specific subgroup meta‐analysis within PSS studies.

Subgroup analyses were conducted across 12 studies using PSS to explore sources of between‐study heterogeneity. By geographical region, pooled prevalence estimates ranged from 62.15% (95% CI: 33.18–91.12) in Africa to 82.32% (95% CI: 70.34–94.30) in Asia, with a borderline significant difference across regions (*p* = 0.05). When grouped by country income level, pooled prevalence was similar between studies from high‐income countries (75.35%, 95% CI: 64.61–86.09) and middle‐income countries (74.17%, 95% CI: 58.63–89.72), with no evidence of subgroup differences (*p* = 0.90). Across COVID‐19 periods, pooled estimates were 53.05% (95% CI: 3.09–103.01) for pre‐COVID studies, 81.33% (95% CI: 70.68–91.99) during COVID, and 74.59% (95% CI: 55.29–93.88) post‐COVID, with no statistically significant differences (*p* = 0.49). Prevalence did not differ significantly by PSS version (*p* = 0.52). However, clinical stage showed a significant subgroup effect, with studies including only clinical‐year students reporting a pooled prevalence of 99.75% (95% CI: 99.40–100.10) compared with 69.29% (95% CI: 55.62–82.97) in samples combining clinical and preclinical students (*p* < 0.001) (Figure 6).

**FIGURE 7:**
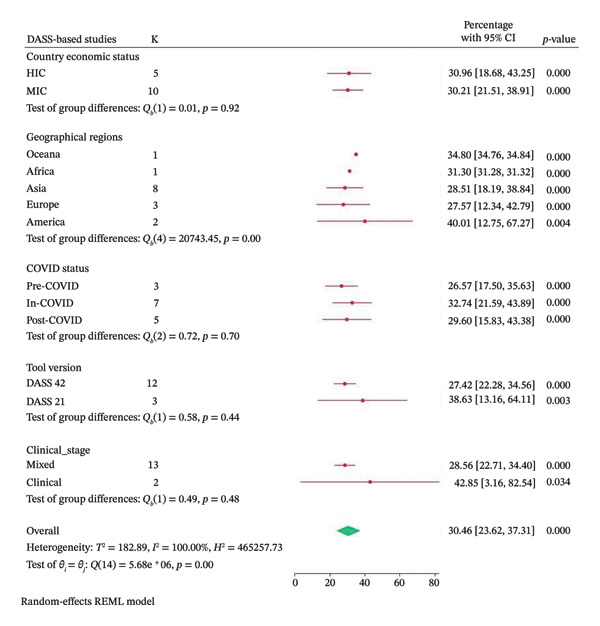
Instrument‐specific subgroup meta‐analysis within DASS studies.

Subgroup analyses were conducted for the 15 studies using the DASS‐Stress subscale. By geographical region, pooled prevalence estimates ranged from 27.57% (95% CI: 15.13–40.00) in Europe to 40.01% (95% CI: 20.73–59.29) in the Americas, with a statistically significant difference across regions (*p* < 0.001). When grouped by country income level, pooled prevalence was similar between high‐income countries (30.96%, 95% CI: 19.98–41.95) and low‐income countries (30.21%, 95% CI: 21.96–38.46), with no evidence of subgroup differences (*p* = 0.91). Across COVID‐19 periods, pooled estimates were 26.57% (95% CI: 19.17–33.97) pre‐COVID, 32.74% (95% CI: 22.42–43.06) during COVID, and 29.60% (95% CI: 17.28–41.92) post‐COVID, with no significant differences (*p* = 0.63). Prevalence did not differ significantly by DASS version (*p* = 0.35) or by clinical stage (*p* = 0.33) (Figure 7).

### 3.7. Instrument‐Specific Sensitivity Analyses

**FIGURE 8:**
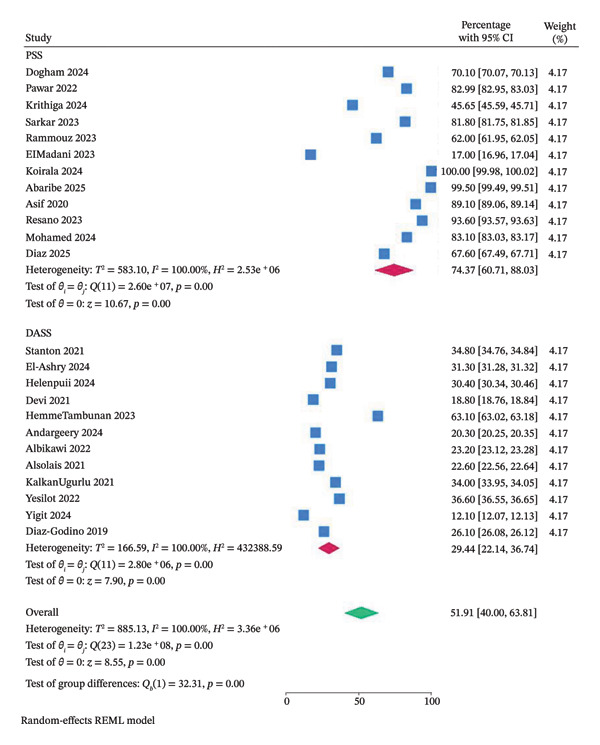
Sensitivity analysis of instrument‐specific pooled prevalence for moderate‐to‐high stress within studies using PSS and DASS tools.

Sensitivity analyses were conducted to assess the robustness of pooled prevalence estimates when restricted to studies with moderate and low risk of bias. Among PSS‐based studies meeting this criterion (*n* = 12), the pooled prevalence of moderate‐to‐high stress was 74.37% (95% CI: 61.29–87.45), with substantial heterogeneity. For DASS‐based studies with moderate or low risk of bias (*n* = 12), the pooled prevalence was 29.44% (95% CI: 22.14–36.74), accompanied by high heterogeneity (Figure 8). The consistency of findings despite exclusion of high‐risk studies suggests that observed stress levels are unlikely to be entirely artefactual; however, the magnitude of prevalence may still be overestimated.

### 3.8. Instrument‐Specific Meta‐Regression Analyses

Meta‐regression analyses were conducted separately for PSS‐based and DASS‐based studies to examine whether study‐level characteristics accounted for between‐study variability in prevalence estimates. For PSS studies, none of the examined covariates—including geographical region (*p* = 0.253), country income status (*p* = 0.607), COVID‐19 period (*p* = 0.565), PSS version (*p* = 0.865), or study quality (*p* = 0.984)—were significantly associated with prevalence. The clinical stage showed a borderline association (coefficient = 30.78, *p* = 0.063) but did not reach statistical significance. For DASS studies, no covariates demonstrated significant associations with prevalence, including geographical region (*p* = 0.660), country income status (*p* = 0.661), COVID‐19 period (*p* = 0.533), DASS version (*p* = 0.256), clinical stage (*p* = 0.250), or study quality (*p* = 0.930). Overall, the meta‐regression models did not identify any study‐level predictors that explained the substantial heterogeneity observed within either instrument (Table 2).

**TABLE 2:** Instrument‐specific meta‐regression analyses across PSS and DASS studies.

Instrument	Coefficient	SE	*z*	*p* value	95% conf. interval
*PSS Tool*					
Geographical regions	11.63409	10.187	1.14	0.253	−8.33–31.60
Country income status	10.92617	21.241	0.51	0.607	−30.70–52.55
COVID status	6.207033	10.784	0.58	0.565	−14.93–27.34
Tool version	3.988454	23.483	0.17	0.865	−42.03–50.01
Clinical stage	30.78409	16.575	1.86	0.063	−1.70–63.27
NOS study quality	−0.2816781	14.284	−0.02	0.984	−28.27–27.71
_cons	20.06649	46.688	0.43	0.667	−71.44–111.57

*DASS Tool*					
Geographical regions	1.480078	3.368128	0.44	0.660	−5.12–8.08
Country income status	3.119583	7.106382	0.44	0.661	−17.04–10.80
COVID status	2.762978	4.428114	0.62	0.533	−5.91–11.44
Tool version	10.21605	8.997941	1.14	0.256	−7.41–27.85
Clinical stage	11.00586	9.564289	1.15	0.250	−7.73–29.75
NOS study quality	0.4279337	4.83999	0.09	0.930	−9.91–9.05
_cons 20.06649	21.52056	15.13051	1.42	0.155	−8.13–51.17

### 3.9. Publication Bias

**FIGURE 9:**
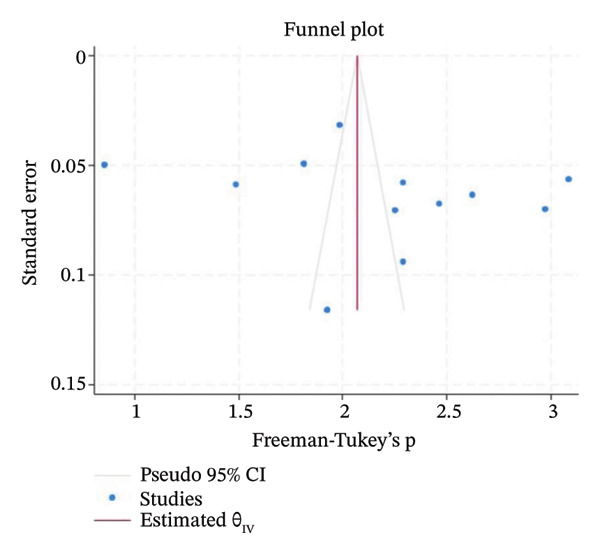
Funnel plot assessing publication bias for PSS‐based studies.

**FIGURE 10:**
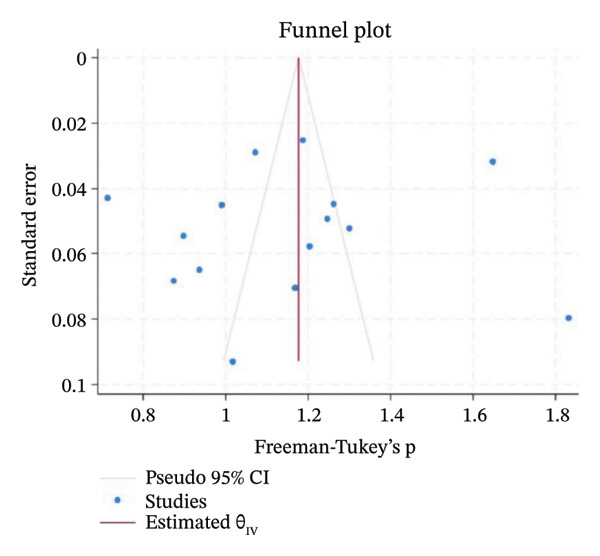
Funnel plot assessing publication bias for DASS‐based studies.

**FIGURE 11:**
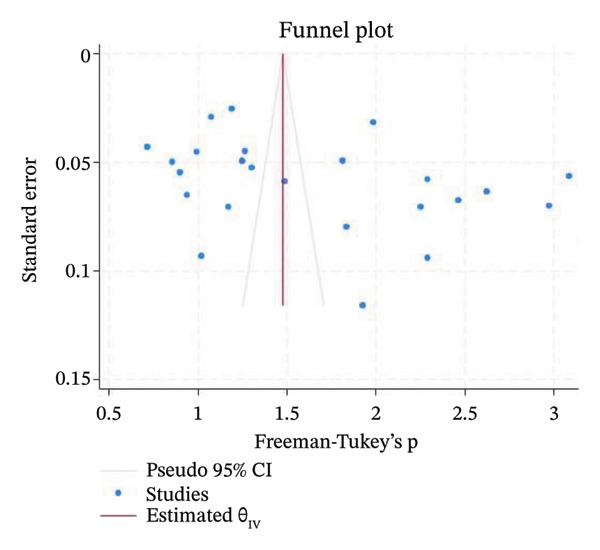
Funnel plot assessing publication bias with moderate‐ and low‐risk‐of‐bias studies.

**FIGURE 12:**
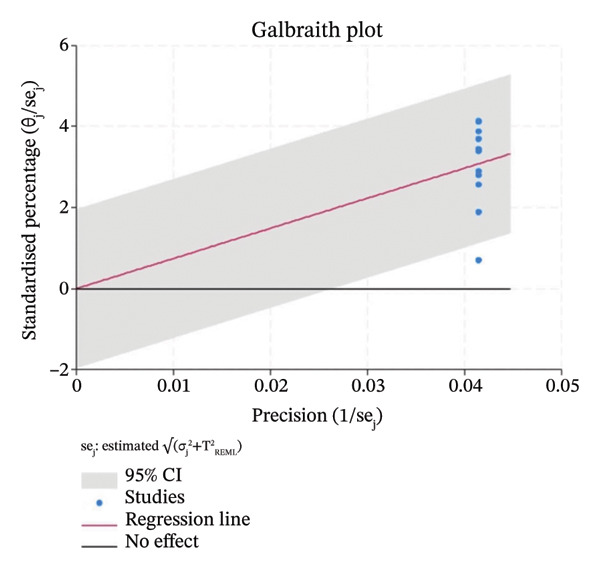
Galbraith plot for PSS‐based studies.

**FIGURE 13:**
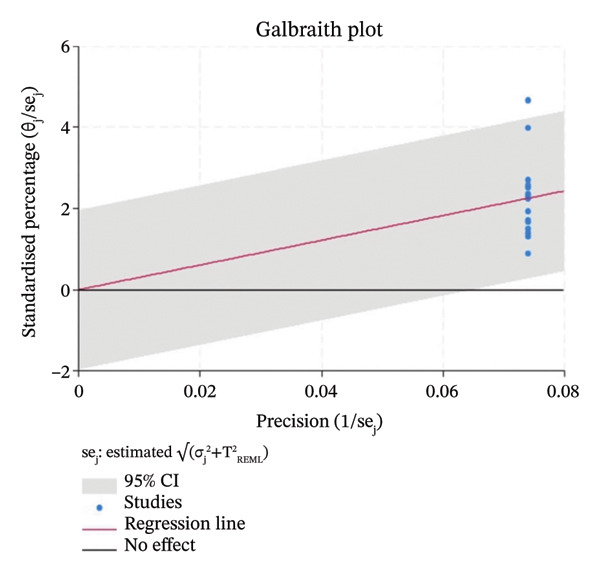
Galbraith plot for DASS‐based studies.

Publication bias was assessed using funnel plots for studies employing the PSS and DASS instruments. Visual inspection of both plots showed some asymmetrical distributions of study estimates around the pooled effect size, suggesting the possibility of small‐study effects. Begg’s test did not suggest significant publication bias for either instrument, with *p* = 0.1499 for PSS studies and *p* = 0.691 for DASS studies. Overall, there was no strong statistical or visual evidence of publication bias (Figures 9 and 10). The funnel plot for studies with low and moderate risk of bias is presented in Figures 11, 12 and 13. This interpretation is supported by analyses restricted to studies with low‐to‐moderate risk of bias, which showed consistent patterns.

### 3.10. Narrative Synthesis of Studies Not Included in Meta‐Analysis

Three cross‐sectional studies, **89, 90 and 91** were not included in the quantitative meta‐analysis due to the use of noncomparable stress assessment instruments and/or incompatible outcome reporting. These studies were conducted across Egypt, Iran and China, all classified as middle‐income countries, with sample sizes ranging from 200 to 1538 undergraduate nursing students. Two studies employed convenience sampling, while one used stratified random sampling, and response rates were consistently high (> 90%). All studies included students across Years 1–4 and enrolled mixed‐gender cohorts, with two conducted in the pre‐COVID‐19 period and one in the post‐COVID‐19 period.

Stress was assessed using three different instruments: the PSS‐14, the Academic Stress Scale‐27, and the Student Nurse Stress Index (SINS‐CN‐43) [88–90]. Due to substantial conceptual and scoring differences across these tools, pooled prevalence estimates could not be derived. The Egyptian study using the PSS‐14 reported predominantly low perceived stress levels, whereas the Iranian post‐COVID study employing the Academic Stress Scale‐27 similarly identified low levels of academic stress among nursing students. In contrast, the Chinese study using the SINS‐CN‐43 reported moderate stress levels, suggesting a comparatively higher burden when stress was measured using a nursing‐specific instrument. Overall, while methodological heterogeneity precluded quantitative synthesis, these studies consistently indicate the presence of stress among undergraduate nursing students, with observed variations likely reflecting instrument sensitivity, contextual factors and training period.

## 4. Discussion

This systematic review of 42 cross‐sectional studies demonstrates an instrument‐defined moderate‐to‐high stress across diverse educational settings and contexts. When reported as a single descriptive summary across instruments, the pooled prevalence of stress was 49.98%. Whilst this is a useful indicator of overall burden, it should be interpreted strictly descriptively since the estimate aggregates non‐equivalent instruments measuring different stress constructs (perceived appraisal vs. symptom‐based stress). In contrast, instrument‐specific analyses demonstrate divergent estimates. Studies using the PSS had a pooled prevalence of moderate‐to‐high stress of 74.37% and a pooled mean score of 20.93, reflecting a moderate level of stress. Studies using the DASS‐Stress subscale yielded a pooled prevalence of 30.46% and a pooled mean of 10.39. All pooled estimates were accompanied by extreme between‐study heterogeneity (*I* ^2^ ≈ 100% for prevalence pools; *I* ^2^ > 99% for mean scores). Accordingly, the most defensible conclusions come from the instrument‐specific syntheses rather than the cross‐instrument aggregate. These findings from PSS‐based and DASS‐based analyses should not be interpreted as directly comparable, and differences between them likely reflect underlying conceptual distinctions rather than true differences in stress burden.

Consistent with this conceptual framing, we intentionally synthesised prevalence estimates separately by measurement instrument rather than assuming cross‐instrument equivalence: We pooled instrument‐defined moderate‐to‐high stress for studies using the PSS and the DASS‐Stress subscale and probed between‐study variability with subgroup analyses and meta‐regression to capture methodological and contextual sources of heterogeneity. Substantial between‐study heterogeneity was evident across pooled analyses, reflecting wide variation in populations, settings, and what each instrument measures; accordingly, pooled prevalences are presented as descriptive summaries of the overall burden of instrument‐defined stress rather than precise population parameters. Importantly, meta‐regression did not identify robust study‐level predictors of residual heterogeneity, and sensitivity analyses restricted to studies at moderate or low risk of bias yielded similar instrument‐specific estimates.

Despite stratification by instrument, substantial heterogeneity persisted, suggesting that heterogeneity in reported stress prevalence is not solely attributable to the choice of measurement tool but reflects deeper variability in study contexts, populations and operationalisation of stress. Importantly, the instrument‐specific approach in this review does not aim to minimise heterogeneity, but rather to identify its sources. By separating perceived stress from affective stress symptomatology, this analysis demonstrates that variability in prevalence estimates is partly measurement‐driven and not indicative of a single, stable underlying construct. These findings highlight a fundamental limitation in attempts to synthesise stress prevalence across heterogeneous instruments and suggest that stress should be interpreted as a multidimensional construct rather than a unified epidemiological measure operationalised by various explored and unexplored methodological and contextual factors. Moreover, the meta‐regression findings should be interpreted with caution due to a small number of studies and study‐level covariates, limiting power and introducing ecological constraints; thus, the results are best considered exploratory.

Previous syntheses of nursing‐student stress have documented the high and variable burden of stress but have differed in scope and methods. Several recent reviews and meta‐analyses quantified prevalence or symptom severity but tended to pool across diverse instruments, focusing on certain periods, for example, pandemic studies or regions [30, 47–49]. For instance, an overview of systematic reviews in the Middle East and North Africa highlighted wide prevalence ranges and largely narrative syntheses across reviews [1]. Major meta‐analytic attempts have since estimated pooled stress levels globally, but typically aggregated heterogeneous measures of stress, limiting interpretability with respect to what aspect of ‘stress’ is being measured [9, 30]. COVID‐era meta‐analyses have further documented elevated mental‐health problems during the pandemic but, likewise, emphasised the overall prevalence of stress symptoms rather than instrument‐specific constructs [91]. While the findings highlight the multidimensional nature of stress, the reported estimates reflect instrument‐specific constructs rather than a unified measure; therefore, any interpretation of a ‘global burden’ should be approached cautiously.

The substantial variability we observed across studies plausibly reflects differences in educational context rather than a single underlying cause: factors such as heavy academic workload and high‐stakes assessment pressure, the intensity and quality of clinical exposure (including fear of making errors and role uncertainty), and the organisation of clinical learning environments all vary between programmes and are commonly reported stress drivers in the nursing‐education literature [92, 93]. Disruptions related to the COVID‐19 pandemic, including curtailed or altered clinical placements, rapid pivots to remote learning, and heightened infection concerns among students, likely amplified those contextual pressures in some settings but not others, producing time‐period effects that are heterogeneous across countries and cohorts [43, 93]. Finally, regional and health‐system differences (and correlated income‐level factors) shape placement opportunities, supervision, and coping resources available to students, which can further modify measured prevalence; thus, the observed between‐study heterogeneity should be interpreted as an ecological pattern arising from multiple, interacting contextual features rather than evidence of a single causal driver [94].

From an educational perspective, the consistently high prevalence of instrument‐defined moderate‐to‐high perceived stress observed in this review has important implications for learning, retention and student well‐being. Elevated stress among nursing students has been associated with poorer academic engagement, impaired clinical learning, burnout symptoms, and increased intention to leave programmes, all of which pose risks to educational quality and future workforce sustainability [42, 95, 96]. These findings underscore the need for nursing programmes to move beyond reactive, individual‐level responses and adopt proactive, context‐sensitive strategies that recognise stress as a structural and educational issue rather than solely a personal vulnerability.

In practice the conceptual differences between PSS and the DASS Stress subscale highlight the importance of selecting tools aligned with the intended purpose of assessment. PSS is well suited for routine monitoring of nursing students’ perceived stress, as it captures cognitive appraisal of stressors encountered during everyday academic demands as well as high‐pressure periods such as clinical placement transitions and assessment‐intensive weeks. Elevated PSS scores not only reflect heightened exposure to stressors, including workload, clinical demands, time pressure and assessment load, but may also signal reduced coping capacity and lower resilience. In contrast, the DASS‐Stress subscale assesses symptom‐based stress, capturing physiological and affective manifestations such as tension, irritability, difficulty relaxing and autonomic arousal, and is potentially useful to identify students at risk with severity of symptoms. Subgroups consistently showing higher stress, such as students in clinical stages or those studying during disruptive periods like COVID‐19, may benefit from targeted institutional strategies focused on stress management, resilience building, mindfulness, coping skills and mental health promotion [96, 97]. Embedding brief tools such as the PSS into programme‐level monitoring could support earlier identification of at‐risk students and enable timely, tailored support. A dual‐measurement framework may therefore provide a more comprehensive basis for evaluating student well‐being initiatives and tailoring interventions to both stress appraisal and symptom severity over time.

Key strengths of this review include a comprehensive and a methodologically rigorous, instrument‐specific analytic strategy with planned subgroup, sensitivity, and meta‐regression analyses to probe heterogeneity. The consistency of findings across studies with low‐to‐moderate risk of bias strengthens confidence in the robustness of the pooled estimates. These features increase confidence that the review maps the contemporary and global evidence base and avoids the common pitfall of aggregating non‐equivalent measures while exploring real‐world variability across settings. The review had several limitations. The very high heterogeneity indicates substantial variability across studies, making the pooled estimates difficult to interpret. Although extensive subgroup analyses and meta‐regression were conducted (including instrument type, COVID‐19 period, training stage and regional and economic classifications), none of these factors explained the between‐study variability. Important contributors such as institutional culture, academic workload, sampling procedures, and unmeasured psychosocial factors were not captured in the reported data. Hence, the pooled estimates should be interpreted with caution, and their reliability and generalisability considered limited. Almost all primary studies were cross‐sectional, precluding inference about trajectories or causality; unstable CIs for some subgroups; and instrument non‐equivalence limits direct comparability across scales. In addition, the limited explanatory power of meta‐regression suggests that commonly reported study‐level variables may be insufficient to capture the complexity of stress experiences across training environments. The limited number of studies within subgroups may have reduced statistical power to detect significant associations. Restricting the review to studies published in English may have introduced potential language bias, as relevant evidence from non‐English publications could not be captured. Another limitation of this review is the use of different stress measurement instruments with non‐equivalent severity thresholds. The PSS and DASS assess related but distinct constructs and apply different categorisation frameworks, limiting direct comparability of prevalence estimates. Although analyses were conducted separately for each instrument, a formal sensitivity analysis using harmonised thresholds was not feasible due to inconsistent reporting of category‐level data across studies. As a result, residual measurement heterogeneity may influence the observed variation in prevalence. The inclusion of different PSS versions (primarily PSS‐10 and one PSS‐29), despite using study‐specific cut‐offs, may introduce measurement heterogeneity and limit direct comparability of pooled estimates. Together, these strengths and limitations mean the review robustly describes the burden of instrument‐defined stress but cannot definitively attribute sources or time trends.

To lead the field forward, this review demonstrates that instrument‐level stratification improves conceptual clarity, even when statistical heterogeneity remains high. The future research should prioritise longitudinal cohort studies that map stress trajectories through key educational transitions (entry, first clinical placement, final year) and link trajectories to outcomes (academic performance, attrition, well‐being). Parallel priorities include development and adoption of a standardised measurement framework that includes both perceived‐appraisal measures and symptom‐based instruments or validated cross‐walks/calibration studies to allow harmonised pooling. There is a need for rigorous mixed‐methods research to contextualise quantitative trends. Adequately powered cluster‐randomised or stepped‐wedge trials of curriculum‐embedded, context‐sensitive interventions are also required. Greater inclusion of low‐ and middle‐income settings will help clarify system‐level drivers. Investing in these designs will help move the field beyond descriptive prevalence mapping towards generating causal and implementation evidence that can inform educational practice.

In conclusion, the findings provide a contemporary, instrument‐specific meta‐analysis representing a key contribution, offering a more accurate and conceptually aligned synthesis of stress burden. This helps to inform programme‐level monitoring and development of context‐sensitive well‐being strategies among nursing students. Notably, PSS and DASS measure overlapping but distinct constructs; their pooled estimates should be compared with caution. This highlights the need for future research to better capture unmeasured contextual, methodological and institutional factors contributing to the substantial heterogeneity.

### 4.1. Relevance to Clinical Practice

This review highlights the substantial burden of moderate‐to‐high stress among undergraduate nursing students and demonstrates that prevalence estimates vary markedly by measurement instrument and should be interpreted within their conceptual frameworks with caution. Recognising these instrument‐specific differences is essential for clinicians, educators and well‐being teams when interpreting stress data and designing curricula and support strategies. The findings reinforce the need for measurement‐aware monitoring, targeted interventions during high‐risk training stages, and the integration of consistent, theory‐aligned stress assessment tools in clinical education settings. Moreover, it will enable early screening for stress‐related mental health challenges during training.

## Author Contributions

Md Nazmul Karim, Michelle Lazarus, Dragan Ilic, Zahra Aziz and Sibgha Fatima contributed to the development of the study design. Sibgha Fatima performed the search strategies and data analysis. Sibgha Fatima and Carol Tra Vu piloted the data extraction form and performed risk‐of‐bias assessments. Md Nazmul Karim performed as the 3rd reviewer to resolve any disagreement. Sibgha Fatima and Md Nazmul Karim contributed to the preparation of the manuscript.

## Funding

The Article Processing Charge (APC) for this manuscript was covered by Monash University Australia through the CAUL agreement with the publisher. This research was supported by the Commonwealth through an Australian Government Research Training Program Scholarship https://doi.org/10.82133/C42F-K220. Open access publishing was facilitated by Monash University as part of the Wiley–Monash University agreement via the Council of Australasian University Librarians.

## Disclosure

All authors read and reviewed the final manuscript.

## Ethics Statement

This review adhered to PRISMA 2020 reporting guidelines, and ethical approval was not required as only de‐identified, published data were used.

## Consent

No individual patient data or identifiable information are included, consistent with PRISMA 2020 guidelines.

## Conflicts of Interest

The authors declare no conflicts of interest.

## Supporting Information

Additional supporting information can be found online in the Supporting Information section.

## Supporting information


**Supporting Information** Supporting Appendix A: Search strategy for all databases, study quality assessment using the Newcastle–Ottawa Scale (NOS), and pooled prevalence tables for all included studies.

## Data Availability

The data that support the findings of this study are available from the corresponding author upon reasonable request.
