# Supplementary material for: Mapping the Multidimensional Stress Burden in Nursing Students: A Systematic Review and Instrument‐Specific Meta‐Analysis
Source: Nurs Res Pract. 2026 Jul 12;2026:5514459. doi: 10.1155/nrp/5514459 (PMC13358211; doi:10.1155/nrp/5514459)
Supplement: Supplementary file 1 — Supporting Information Supporting Appendix A: Search strategy for all databases, study quality assessment using the Newcastle–Ottawa Scale (NOS), and pooled prevalence tables for all included studies. [file NRP-2026-5514459-s001.docx]

**Supplementary Appendix A**

**Table S1.** **Full search strategy for all databases.**

Ovid MEDLINE(R) <1946 to December 12, 2025>

| **#** | **Query** | **Results from 15 Dec 2025** |
| --- | --- | --- |
| 1 | students, nursing/ | 34,797 |
| 2 | (nursing students or undergraduate nursing students or baccalaureate students in nursing or baccalaureate nursing).mp. | 21,904 |
| 3 | stress, psychological/ or burnout, psychological/ or emotional exhaustion/ or financial stress/ or subjective stress/ or time pressure/ | 150,496 |
| 4 | (perceived stress* or student stress* or subjective stress or academic stress* or psychologic* stress*).mp. | 25,150 |
| 5 | 3 or 4 | 160,888 |
| 6 | incidence/ or prevalence/ | 681,927 |
| 7 | (incidence or prevalence or epidemiology or distribution or proportion or magnitude).mp. [mp=title, book title, abstract, original title, name of substance word, subject heading word, floating sub-heading word, keyword heading word, organism supplementary concept word, protocol supplementary concept word, rare disease supplementary concept word, unique identifier, synonyms, population supplementary concept word, anatomy supplementary concept word] | 4,810,721 |
| 8 | 6 or 7 | 4,810,721 |
| 9 | 1 or 2 | 39,765 |
| 10 | 5 and 8 and 9 | 224 |
| 11 | limit 10 to yr="2010 -Dec 2025" | 179 |

**Database 2**
Ovid Emcare <1995 to 2025 Week 48>

| **#** | **Query** | **Results from 15 Dec 2025** |
| --- | --- | --- |
| 1 | nursing education/ or nursing students/ | 58,624 |
| 2 | (Nursing student s or baccalaureate nursing students or graduate nursing students or male nursing students or female nursing students).mp. | 1,910 |
| 3 | stress/ or academic stress/ or financial strain/ or perceived stress/ or burnout/ or coping style/ or distress/ or emotional exhaustion/ or "stress and trauma related disorders"/ or stress management/ | 119,609 |
| 4 | (prevalence or incidence or epidemiology).mp. [mp=title, abstract, heading word, drug trade name, original title, device manufacturer, drug manufacturer, floating subheading word, device trade name, keyword heading word, candidate term word] | 993,756 |
| 5 | cross sectional [studies.mp](http://studies.mp/). | 11,615 |
| 6 | cohort [studies.mp](http://studies.mp/). | 23,680 |
| 7 | school stress/ or mental stress/ or test anxiety/ | 30,450 |
| 8 | mental stress/ or behavioral stress/ or cognitive stress/ or emotional stress/ or family stress/ or interpersonal stress/ or life stress/ or time pressure/ | 56,777 |
| 9 | burnout/ or student burnout/ | 21,450 |
| 10 | [stress.mp](http://stress.mp/). | 441,380 |
| 11 | 1 or 2 | 59,230 |
| 12 | 3 or 7 or 8 or 9 or 10 | 491,030 |
| 13 | 4 or 5 or 6 | 1,015,824 |
| 14 | 11 and 12 and 13 | 319 |
| 15 | limit 14 to yr="2010 -Dec 2025" | 295 |

**Database 3**
APA PsycInfo <1806 to December 2025 Week 2>

| **#** | **Query** | **Results from 15 Dec 2025** |
| --- | --- | --- |
| 1 | nursing education/ or nursing students/ | 13,936 |
| 2 | (Nursing student s or baccalaureate nursing students or graduate nursing students or male nursing students or female nursing students).mp. | 1,078 |
| 3 | academic stress/ or burnout/ or stress/ or academic achievement motivation/ or school adjustment/ or social stress/ | 109,709 |
| 4 | stress/ or academic stress/ or financial strain/ or perceived stress/ or burnout/ or coping style/ or distress/ or emotional exhaustion/ or "stress and trauma related disorders"/ or stress management/ | 139,703 |
| 5 | [stress.mp](http://stress.mp/). | 359,269 |
| 6 | 3 or 4 or 5 | 405,003 |
| 7 | (prevalence or incidence or epidemiology).mp. [mp=title, abstract, heading word, table of contents, key concepts, original title, tests & measures, mesh word] | 262,993 |
| 8 | cross sectional [studies.mp](http://studies.mp/). | 77,316 |
| 9 | 1 or 2 | 14,091 |
| 10 | 3 or 4 or 5 | 405,003 |
| 11 | 7 or 8 | 318,690 |
| 12 | 9 and 10 and 11 | 146 |
| 13 | limit 12 to yr="2010 -Dec 2025" | 129 |

**Database 4**
Embase Classic+Embase <1947 to 2025 December 11>

| **#** | **Query** | **Results from 15 Dec 2025** |
| --- | --- | --- |
| 1 | nursing student/ | 39,407 |
| 2 | nursing student/ or baccalaureate nursing student/ or graduate nursing student/ or male nursing student/ | 39,602 |
| 3 | mental stress/ | 116,597 |
| 4 | emotional stress/ or mental stress/ | 154,967 |
| 5 | school stress/ or test anxiety/ | 1,126 |
| 6 | financial stress/ or financial distress/ | 7,880 |
| 7 | mental stress/ or life stress/ or subjective stress/ or time pressure/ | 123,372 |
| 8 | physically induced stress/ or transport stress/ | 498 |
| 9 | academic stress*.mp. | 1,278 |
| 10 | perceived stress*.mp. | 24,441 |
| 11 | psychologic*[stress.mp](http://stress.mp/). | 24 |
| 12 | 3 or 4 or 5 or 6 or 7 or 8 or 9 or 10 or 11 | 187,819 |
| 13 | (incidence or prevalence or epidemiology or distribution or proportion or magnitude).mp. | 7,125,065 |
| 14 | cross-sectional study/ or epidemiology/ or methodology/ | 2,786,477 |
| 15 | cohort analysis/ or methodology/ or statistical analysis/ | 3,456,020 |
| 16 | (cohort stud* or cohort analysis or cross-sectional stud*).mp. | 2,393,189 |
| 17 | 1 or 2 | 39,602 |
| 18 | 13 or 14 or 15 | 10,216,811 |
| 19 | 12 and 17 and 18 | 540 |
| 20 | limit 19 to yr="2010 -Dec 2025" | 445 |

**Table S2. Study Quality assessment using the Newcastle–Ottawa Scale (NOS).**

| **Author_**Year | **NOS_Selection_score( /5)** | **NOS_Comparibilty_score ( /2)** | **NOS_Outcome_score ( /3)** | **NOS_Total_score ( /10)** | **NOS_Quality_category** |
| --- | --- | --- | --- | --- | --- |
| Ezo 2024 | 5 | 2 | 3 | 10 | low |
| Smith 2017 | 2 | 1 | 3 | 6 | moderate |
| Asif 2020 | 4 | 0 | 1 | 5 | moderate |
| Nazari 2025 | 4 | 2 | 3 | 9 | low |
| Farber 2025 | 4 | 0 | 3 | 7 | low |
| Pawar 2022 | 3 | 0 | 2 | 5 | moderate |
| Andargeery 2024 | 4 | 2 | 3 | 9 | low |
| Krithiga 2024 | 5 | 0 | 2 | 7 | low |
| Rammouz 2023 | 4 | 2 | 3 | 9 | low |
| Smith 2022 | 3 | 2 | 3 | 8 | low |
| Baruah 2022 | 2 | 0 | 2 | 4 | high |
| Albikawi 2022 | 3 | 0 | 3 | 6 | moderate |
| Ngoc 2023 | 4 | 2 | 1 | 7 | high |
| Resano 2023 | 4 | 2 | 3 | 9 | low |
| Sarkar 2023 | 4 | 2 | 3 | 9 | low |
| AlMaqbali 2023 | 3 | 0 | 3 | 6 | moderate |
| Helenpuii 2024 | 2 | 0 | 3 | 5 | moderate |
| Dogham 2024 | 3 | 2 | 3 | 8 | low |
| Mohamed 2024 | 4 | 2 | 2 | 8 | low |
| El-Ashry 2024 | 5 | 2 | 3 | 10 | low |
| Cao 2025 | 3 | 0 | 3 | 6 | moderate |
| Komariah 2025 | 3 | 0 | 3 | 5 | moderate |
| Sarfika 2025 | 4 | 0 | 2 | 7 | low |
| Abaribe 2025 | 2 | 0 | 3 | 5 | moderate |
| BernierCarney 2025 | 2 | 0 | 2 | 4 | high |
| Amr 2011 | 5 | 2 | 3 | 10 | low |
| Lee 2014 | 4 | 0 | 2 | 6 | moderate |
| Diaz-Godino 2019 | 3 | 0 | 3 | 6 | moderate |
| Onieva-Zafra 2020 | 3 | 2 | 3 | 8 | low |
| KalkanUgurlu 2021 | 3 | 0 | 2 | 5 | moderate |
| Alsolais 2021 | 3 | 0 | 3 | 6 | moderate |
| Stanton 2021 | 4 | 2 | 3 | 10 | low |
| Devi 2021 | 4 | 0 | 2 | 6 | moderate |
| Yesilot 2022 | 4 | 2 | 3 | 9 | low |
| ElMadani 2023 | 3 | 0 | 3 | 6 | moderate |
| AlMaqbali 2023 | 3 | 0 | 3 | 6 | moderate |
| HemmeTambunan 2023 | 3 | 0 | 2 | 5 | moderate |
| Stubin 2024 | 2 | 0 | 2 | 4 | high |
| Yigit 2024 | 3 | 0 | 2 | 5 | moderate |
| Koirala 2024 | 5 | 0 | 2 | 7 | low |
| Diaz 2025 | 2 | 2 | 3 | 7 | low |
| Ayaz-Alkaya 2025 | 2 | 2 | 3 | 7 | low |

**Table S3. Pooled Prevalence across included Studies.**

| **Study** | **Percentage** | **[95% conf.**  **interval]** |
| --- | --- | --- |
| Stanton 2021 | 34.8 | 34.75 - 34.84 |
| Dogham 2024 | 70.1 | 70.07 - 70.12 |
| El-Ashry 2024 | 31.3 | 31.27 - 31.32 |
| Pawar 2022 | 82.99 | 82.94 - 83.03 |
| Krithiga 2024 | 45.65 | 45.59 - 45.70 |
| Sarkar 2023 | 81.8 | 81.74 - 81.85 |
| Baruah 2022 | 17.7 | 17.64 - 17.75 |
| Helenpuii 2024 | 30.4 | 30.33 - -30.46 |
| Devi 2021 | 18.8 | 18.75 - 18.84 |
| HemmeTambunan 2023 | 63.1 | 63.02 - 63.17 |
| Rammouz 2023 | 62 | 61.95 - 62.04 |
| ElMadani 2023 | 17 | 16.96 - 17.03 |
| Koirala 2024 | 100 | 99.98 - 100.02 |
| Abaribe 2025 | 99.5 | 99.49 - 99.51 |
| Asif 2020 | 89.1 | 89.06 - 89.13 |
| Resano 2023 | 93.6 | 93.57 - 93.63 |
| Mohamed 2024 | 83.1 | 83.03 - 83.16 |
| Andargeery 2024 | 20.3 | 20.24 - 20.35 |
| Albikawi 2022 | 23.2 | 23.12 - 23.27 |
| Alsolais 2021 | 22.6 | 22.56 - 22.63 |
| KalkanUgurlu 2021 | 34 | 33.95 - 34.04 |
| Yesilot 2022 | 36.6 | 36.55 - 36.64 |
| Yigit 2024 | 12.1 | 12.07 - 12.12 |
| Diaz 2025 | 67.6 | 67.49 - 67.70 |
| Stubin 2024 | 53.92 | 53.88 - 53.95 |
| Ngoc 2023 | 32 | 31.94 - 32.05 |
| Diaz-Godino 2019 | 26.1 | 26.07 - 26.12 |
| theta | 49.96 | 39.25 - 60.70 |

tau^2^ = 839.5287, I^2^ (%) = 100.00, H^2^ = 3.1e+06
